# Regulation of RNA stability at the 3′ end

**DOI:** 10.1515/hsz-2020-0325

**Published:** 2020-11-27

**Authors:** Mallory I. Frederick, Ilka U. Heinemann

**Affiliations:** Department of Biochemistry, Schulich School of Medicine and Dentistry, The University of Western Ontario, London ON, Canada

**Keywords:** microRNA, mixed tails, mRNA, nucleotide addition, RNA degradation, tRNA

## Abstract

RNA homeostasis is regulated by a multitude of cellular pathways. Although the addition of untemplated adenine residues to the 3′ end of mRNAs has long been known to affect RNA stability, newly developed techniques for 3′-end sequencing of RNAs have revealed various unexpected RNA modifications. Among these, uridylation is most recognized for its role in mRNA decay but is also a key regulator of numerous RNA species, including miRNAs and tRNAs, with dual roles in both stability and maturation of miRNAs. Additionally, low levels of untemplated guanidine and cytidine residues have been observed as parts of more complex tailing patterns.

## Introduction

Cellular rates of RNA synthesis and degradation are among the most important factors for gene regulation. While the central dogma of molecular biology states quite simply that genetic information flows from DNA to RNA to proteins, the complex regulation of these processes is evidence that these systems are much more intricate than anticipated. Each step of the gene expression process is heavily regulated, often with nucleotide additions and modifications dictating the fate of a particular RNA molecule. Terminal nucleotidyltransferases (TENTs) are enzymes capable of adding single or multiple nucleotides to the RNA 3′ end. TENTs can be further subdivided based on nucleotide preference: poly(A) polymerases (PAPs) add untemplated adenines, while terminal uridylyltransferases (TUTases) add untemplated uridines. Although there are a number of mechanisms regulating stability and expression of various RNA types, this review will focus on the regulation of mRNAs, miRNAs, and tRNAs by untemplated 3′ nucleotide addition.

Perhaps the most well-known pathway of mRNA regulation is polyadenylation of the 3′ end: following transcription, untemplated adenosine residues are added to a transcript’s 3′ end by a group of TENTs termed poly(A) polymerases (PAPs) (Laishram 2014), stabilizing them via interactions with poly(A) binding proteins (PABPs) (Goss and Kleiman 2013). mRNA deadenylation is catalyzed by various enzymes in the cytoplasm, primarily the CCR4-NOT and PAN ribonuclease complexes (Meyer et al. 2004). As the transcript is deadenylated, fewer PABPs are able to bind to the 3′ UTR, destabilizing the transcript (Lim et al. 2014). Further, 5′ decapping of mRNA is facilitated by Lsm1-7 in combination with DCP1/2 (Lim et al. 2014; Rissland and Norbury 2009). The combination of these events signals for degradation at the 5′ end by XRN1 and at the 3′ end by the exosome (Meyer et al. 2004).

## RNA uridylation – a pathway to mRNA decay

**Figure 1:**
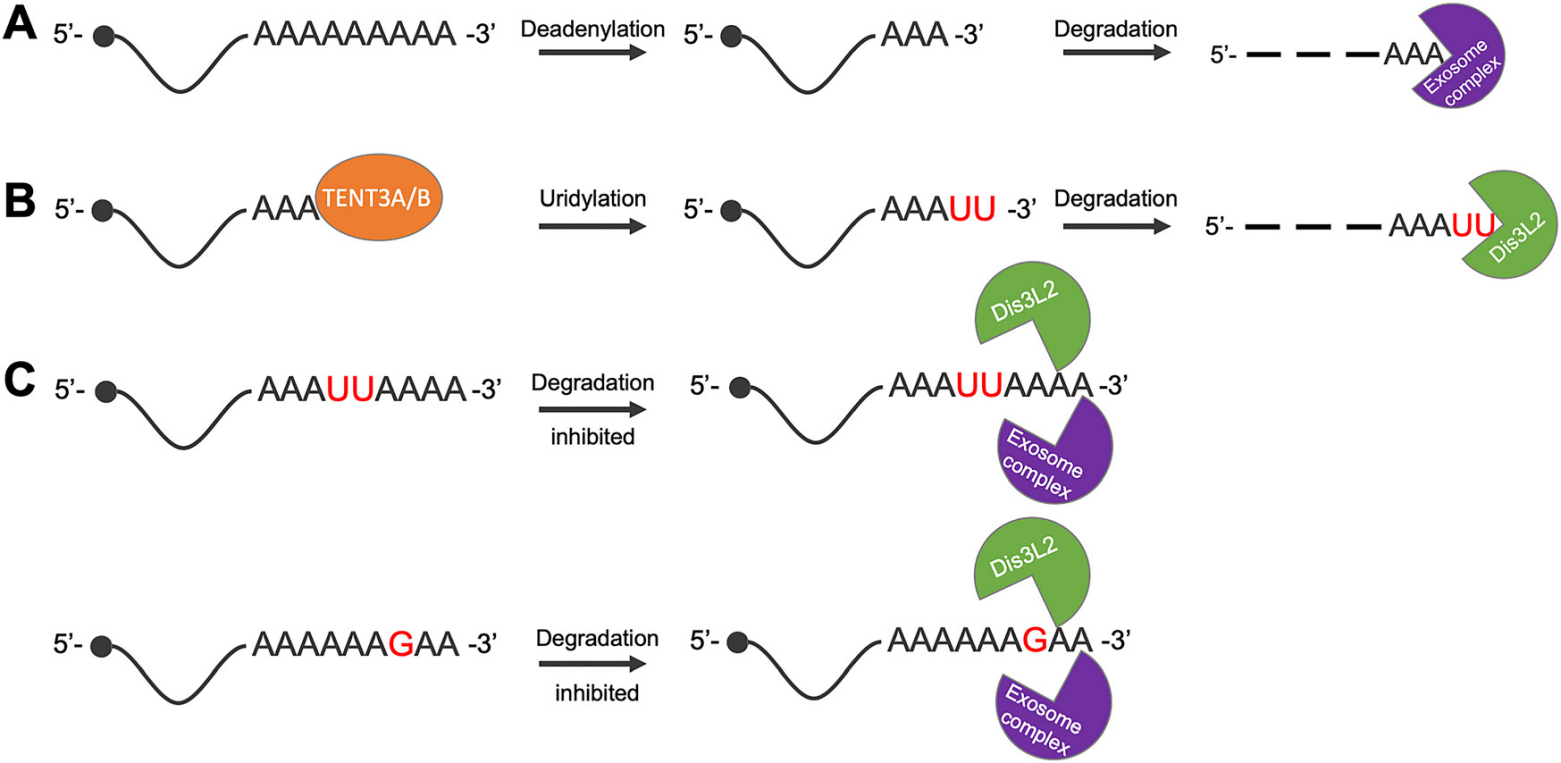
mRNA degradation is regulated by various untemplated nucleotide additions. (A) Canonical degradation of mRNAs occurs when deadenylation of 3′ poly(A) tails destabilizes transcripts in the cytoplasm. Transcripts are degraded in the 3′-5′ direction by the exosome (purple). (B) mRNAs with short (<25 nt) poly(A) tails may be uridylated by TENT3A/B (orange), marking them for degradation by the 3′-5′ exoribonuclease Dis3L2 (green), independent from the exosome. (C) Mixed tails containing combinations of nucleotides may slow degradation by both Dis3L2 and the exosome. 5′ end pathways that contribute to stability of mRNAs are not shown.

Improved RNA-sequencing technologies have recently identified alternate mRNA degradation pathways, diverging from canonical deadenylation dependent decay. 3′-tail sequencing techniques have identified mRNA tails consisting of not only adenosine residues, but combinations of untemplated uridines, cytosines, and guanosines (Figure 1) (Chung et al. 2019b; Chung et al. 2017). The most common of these – aside from canonical poly(A) tails – is poly(U) tailing (Lim et al. 2018). In this recently discovered pathway, untemplated uridine residues are interpreted as an RNA degradation signal by the cell (Figure 1B). While a number of TENTs have been identified in humans, this activity is most commonly attributed to the terminal uridylyltransferase TENT3A (TUT4, ZCCHC11, PAPD3) and its homolog TENT3B (TUT7, ZCCHC6) (Lim et al. 2018), along with the TENT3A homolog Cid1 (caffeine induced death protein 1) in *Schizosaccharomyces pombe* (Chung et al. 2019b). Following uridylation, the mRNA is decapped by the Lsm1-7 decapping complex and 5′-3′ degradation is initiated by the exosome (Rissland and Norbury 2009). Further, 3′ oligouridylation signals for recruitment of the 3′-5′ exoribonuclease Dis3L2 (DIS3-like exoribonuclease 2) to the RNA, followed by degradation as part of uridylation dependent decay (Figure 1B). However, deletion of Dis3L2 alone does not significantly increase the frequency of uridylated transcripts (Chung et al. 2019b; Malecki et al. 2013), implicating redundancy of this pathway, potentially through minor contributions of other nucleases such as Eri1 (3′-5′ exoribonuclease 1), which has been associated with miRNA homeostasis (Thomas et al. 2012).

Uridylation of histone mRNAs following S phase is perhaps the most well-known example of untemplated uridine additions. Mammalian histone mRNAs are oligouridylated at their 3′ ends, presumably by TENT3A, and subsequently degraded by Eri1 or Dis3L2 (Hoefig and Heissmeyer 2014). Although there is some disagreement over which enzyme is responsible, uridylation of these transcripts is a clear signal for their degradation, as uridylation has been linked to transcript abundance (Chang et al. 2014; Chung et al. 2019b). Further, studies using mammalian and vertebrate models have identified a conserved role for TENT3A/B in uridylation-dependent degradation of maternal mRNAs during early zygotic development (Chang et al. 2018; Morgan et al. 2017). In mice, deletion of TENT3A/B by either knockout or catalytic inactivation leads to infertility, presumably due the loss of this degradation step in turnover of the transcriptome during the maternal to zygotic transition (Morgan et al. 2017). In plants, uridylation is not limited to full length mRNAs, but is also used as a degradation signal for RNA-induced silencing complex (RISC)-cleaved fragments produced by RNAi. Following cleavage of the mRNA, the *Arabidopsis thaliana* TUTases URT1 (UTP:RNA uridylyltransferase 1) and HESO1 (HEN1 suppressor 1) are associated with 3′-end uridylation of 5′-cleavage fragments (Zuber et al. 2018). These uridylated 5′ fragments are subsequently degraded by RICE1/2 (RISC-interacting clearing 3′-5′ exoribonuclease) exoribonucleases (Zhang et al. 2017).

In addition to serving as a degradation signal for transcripts that are no longer required by the cell, uridylation has also been proposed to play a role in innate immune responses: TUTases are able to uridylate transcripts encoding viral proteins, directing them for degradation and reducing overall viral load for the host cells (Le Pen et al. 2018). Similarly, uridylation of mRNA encoding long interspersed nuclear retrotransposition elements (LINE-1 elements) decreases the frequency of translocation, either due to reduced transcript abundance or destabilization of target prime reverse transcription for retrotransposition into the genome (Warkocki et al. 2018). Further, uridylation of LINE-1 elements was shown to be nearly as prevalent as canonical poly(A) tailing in some samples, though TENT3A/B may have differential effects on these tailing events.

## Mixed RNA tails send mixed messages

While the role of uridylation in mRNA decay has been increasingly appreciated since the identification of Dis3L2 in 2013 (Chang et al. 2013; Malecki et al. 2013; Ustianenko et al. 2013), more complex tailing mechanisms have yet to be fully elucidated. Mixed tailing events including 5′-UUAAAA-3′ at the 3′ terminus (Chung et al. 2019b), a single guanosine residue at the 3′ poly(A) terminus (Chang et al. 2014; Lim et al. 2018), and poly(UG) tails (Preston et al. 2019) have been noted. A proposed role for these mixed tails is in slowing degradation by exoribonucleases: a recent study showed that a single guanosine contained in the poly(A) tail transiently stalls 3′-5′ deadenylation at these residues (Lim et al. 2018) (Figure 1C). This mechanism is supported by the substrate promiscuity of TENTs both *in vitro* (Chung et al. 2016; Lim et al. 2018) and *in vivo* (Chung et al. 2019b; Preston et al. 2019).

Despite the identification of various TENTs responsible for creating complex tailing patterns, the mechanism of decay for specially modified RNA tails is poorly understood; it is unclear whether Dis3L2 is responsible for degrading other non-canonical RNA tails. Dis3L2 shows substrate promiscuity, requiring only a short poly(U) sequence to degrade a given transcript. Dis3L2 has been shown to preferentially degrade transcripts with as few as two uridines at the 3′ end, even in the presence of 10-fold higher concentrations of adenylated substrate (Ustianenko et al. 2013), and does not seem to be limited beyond the need for this tail (Chang et al. 2013). This indicates a potential role for Dis3L2 in transcriptome-wide regulation and it may be reasonable to predict that Dis3L2 targets mixed tails so long as there are two uridine residues at the 3′ end.

## Untemplated nucleotide addition in miRNA processing and stability

**Figure 2:**
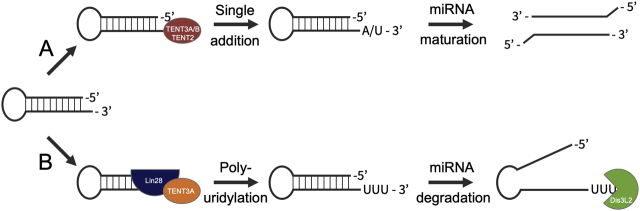
microRNA maturation and degradation is regulated by untemplated nucleotide additions. (A) Pre-miRNAs with 1-nt 3′-overhangs may be monouridylated by TENT3A/B or monoadenylated by TENT2 as part of maturation (red). Once mono-uridylated or -adenylated, the pre-miRNA is processed by DICER/Drosha into the mature miRNA, followed by strand selection and incorporation into the RISC. (B) When recruited by Lin28 (blue), TENT3A (orange) may polyuridylate, rather than monouridylate, pre-miRNAs, suppressing their maturation and processing by DICER/Drosha and marking them for degradation by the U-specific 3′-5′ exoribonuclease Dis3L2 (green).

In addition to targeting mRNA, many TENTs are active toward miRNAs. As part of miRNA maturation and degradation, untemplated uridine (Heo et al. 2012; Heo et al. 2009) and adenine (D’Ambrogio et al. 2012) residues are added to miRNA precursors and mature miRNAs (Figure 2). TENT3A/B are required for let-7 maturation, adding an essential uridine residue to pre-miRNA as a prerequisite for Dicer processing (Heo et al. 2012; Kim et al. 2015). TENT3A is also associated with pre-let-7 polyuridylation, a reaction that leads to pre-miRNA degradation and pathogenically lowered let-7 levels (Kim et al. 2015). Published reports suggest that the TENT3A homolog TENT3B can efficiently fulfill TENT3A’s biological function in miRNA maturation, but not polyuridylation (Kim et al. 2015) (Figure 2). Consequently, TENT3A depletion leads to an overall increase in cellular let-7 levels by reducing let-7 degradation without affecting let-7 maturation (Lin and Gregory 2015).

The 3′-terminal poly(A) polymerase TENT2 (Gld2, Papd4, TUT2) recently gained attention as a regulator of miRNA metabolism, after high-throughput sequencing studies revealed untemplated nucleotide additions at the 3′ termini of nearly 40% of miRNAs (Burroughs et al. 2010; Wyman et al. 2011). Recent studies from us (Chung et al. 2016; Chung et al. 2017) and other groups showed that TENT2 adenylates miRNAs to increase their stability (Burns et al. 2011; Burroughs et al. 2010; D’Ambrogio et al. 2012; Peng et al. 2014). In humans, TENT2 is recruited by QKI-7 (protein quaking isoform 7) to stabilize the miRNA miR-122 in the liver and fibroblasts through monoadenylation (D’Ambrogio et al. 2012; Hojo et al. 2020; Katoh et al. 2009), where its depletion significantly lowers miR-122 levels (Gebert et al. 2014). Interestingly, TENT2 activity is regulated by Akt1 catalyzed phosphorylation, providing a first example of the regulation of miRNA metabolism by Akt1 (Chung et al. 2019a). During infection by the Hepatitis C virus (HCV), TENT2 interacts with the HCV core protein and its activity is downregulated, destabilizing miR-122 maturation during viral infection (Kim et al. 2016; Peng et al. 2014).

The impacts of these nucleotide additions are not as well understood as the mechanisms of addition. Uridylation serves an ambiguous role as both a degradation signal and a biogenesis mechanism: for a particular given miRNA, uridylation may play either role in a context-dependent manner, making it difficult to predict a set of hard-and-fast rules for the impact of these untemplated additions. Although it remains unclear why oligo- and mono-uridylation have such contrasting roles in miRNA regulation, structural analyses propose recruitment by Lin28 as a key factor regulating the extent of uridylation (Yamashita et al. 2019). Further, by identifying the interaction interfaces and role of Lin28 in pre-let-7 uridylation, researchers have shown that recruitment of TENT3A/B by Lin28A is essential for effective polyuridylation (Yamashita et al. 2019) (Figure 2B). Thus, monouridylation may be limited to interactions that occur in the absence of Lin28A, such as transient, spatio-temporally based interactions between TENT3A/B and target RNA substrates.

## Regulation of tRNA function and stability

Despite contributing the largest fraction of ncRNA pools aside from ribosomal RNAs, little is known about post-transcriptional addition of untemplated residues to tRNAs in the TENT pathways that play such large roles in regulation of other RNA families, both coding and non-coding. In addition to nucleotide additions, such as the 3′ end CCA addition to tRNAs discussed below, nucleotide modifications are well known determinants of tRNA stability, and tRNAs are heavily modified during maturation (Alexandrov et al. 2006; Motorin and Helm 2010). Nucleotide modifications are well-studied in the context of tRNA stability, where improperly modified tRNAs are subjected to rapid tRNA decay (RTD). In RTD, hypomodified tRNAs are degraded from the 5′ end by the exonucleases XRN1 and RAT1 as part of cellular quality control (Alexandrov et al. 2006; Chernyakov et al. 2008). However, 3′-untemplated nucleotide additions similar to those known to regulate mRNAs and miRNAs have not been thoroughly investigated for potential roles in tRNA regulation. Further, while these synthesis and posttranscriptional processing pathways are well-studied, the process of tRNA decay outside the RTD pathway is largely unknown.

**Figure 3:**
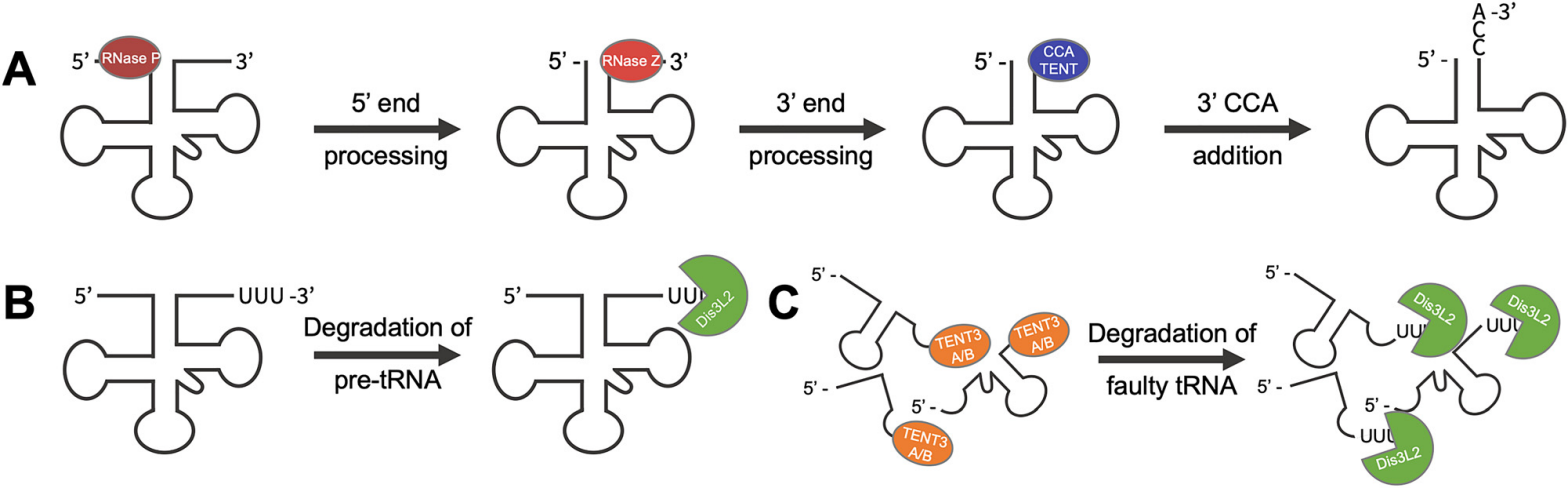
tRNA homeostasis is maintained by multiple processing pathways. (A) tRNA maturation begins following transcription from genomic DNA as a pre-tRNA. Transcripts are cleaved and processed into mature tRNAs in a number of steps, including 5′- and 3′-end processing by RNase P (burgundy) and RNase Z (pink), respectively, and addition of untemplated CCA at the 3′ end for aminoacylation. Faulty tRNAs are degraded via RTD following any of these steps. (B) Improperly processed tRNAs still containing free 3′-uridine residues as part of their trailer sequence may be subjected to uridylation-dependent degradation by Dis3L2 (green). (C) Truncated tRNAs may be uridylated by TENT3A/B (orange) and further degraded by Dis3L2. 5′ end pathways that contribute to stability of tRNAs are not shown.

The tRNA maturation process is composed of various splicing and modification events, including both 5′- and 3′-end processing, intron splicing, and nucleotide modifications (Phizicky and Hopper 2010). As part of 3′ end processing during tRNA maturation, the untemplated nucleotide addition of a 3′-terminal CCA to tRNAs is required for aminoacylation (Figure 3A). Indeed, it has been shown that the CCA adding enzyme plays an important role in tRNA quality control, where a CCACCA tail is added to unstable or damaged tRNAs as a signal for degradation (Betat and Morl 2015). Interestingly, although this addition is catalyzed by a single enzyme – the CCA-adding enzyme – in most eukaryotes, *S. pombe* encodes two separate nucelotidyltransferases responsible for this step of maturation. Two independent studies showed that neither of the nucelotidyltransferases was individually able to rescue a *Saccharomyces cerevisiae* single mutant, but both in combination could restore CCA addition (Preston et al. 2019; Reid et al. 2019).

Recent studies have shown evidence for uridylation and Dis3L2-mediated degradation of tRNAs in two distinct models (Reimao-Pinto et al. 2016; Ustianenko et al. 2016). Following transcription, pre-tRNAs containing their 5′-leader and 3′-trailer sequences may retain a short string of uridines in the 3′-trailer sequences if improperly modified or processed during maturation. These intrinsic polyuridines serve as a signal for degradation by Dis3L2 (Figure 3B). In *Drosophila*, tRNA degradation by Dis3L2 is regulated by a short conserved sequence of uridines in the 3′ trailer sequence approximately 12 nucleotides downstream of the mature 3′ end of these tRNAs (Reimao-Pinto et al. 2016). This is further supported by evidence indicating regulation of tRNA metabolism at the pre-tRNA level, rather than that of the mature tRNA (Alexandrov et al. 2006). A second line of research indicates that truncated or improperly processed tRNAs are oligo- or poly-uridylated, likely by TENT3A in mammals, to serve as a signal for degradation by Dis3L2 (Figure 3C). Here, uridylation is more likely a random, global signal for transcript degradation than a dedicated pathway for degradation of specific families or transcripts. Our lab (Chung et al. 2019b) and others (Lin et al. 2017; Reimao-Pinto et al. 2016; Ustianenko et al. 2016) have shown evidence for this pathway, as tRNAs are confirmed substrates of a number of TENTs. Although the relative contributions of these pathways to tRNA homeostasis remain unclear, tRNAs associate with Dis3L2 both *in vitro* (Chung et al. 2019b) and *in vivo* (Reimao-Pinto et al. 2016; Ustianenko et al. 2016), indicating that uridylation-dependent degradation is a prospective regulator of tRNA homeostasis. Additionally, these models likely coexist, especially given the complexity of uridylation in miRNA biogenesis.

## Conclusion

The complexity of RNA regulation is reflected in the multitude of regulatory pathways, making RNA homeostasis a major topic of research. Among these, interpretation of the functions of untemplated nucleotide additions often remains ambiguous. As we continue to study the mechanisms and regulation of not only various RNAs, but that of TENTs and uridine-specific exoribonucleases such as Dis3L2, the extent and roles of these additions will continue to become clear.
